# *Drosophila* nervous system as a target of aging and anti-aging interventions

**DOI:** 10.3389/fgene.2015.00089

**Published:** 2015-03-10

**Authors:** Leonid V. Omelyanchuk, Mikhail V. Shaposhnikov, Alexey A. Moskalev

**Affiliations:** ^1^Institute of Molecular and Cellular Biology, Siberian Branch of Russian Academy of SciencesNovosibirsk, Russia; ^2^Department of Cytology and Genetics, Novosibirsk State UniversityNovosibirsk, Russia; ^3^Institute of Biology of Komi Science Center of Ural Branch of Russian Academy of SciencesSyktyvkar, Russia; ^4^Moscow Institute of Physics and TechnologyDolgoprudny, Russia; ^5^Engelhardt Institute of Molecular Biology, Russian Academy of SciencesMoscow, Russia

**Keywords:** conditional expression, nervous system, *Drosophila melanogaster*, longevity, aging, anti-aging interventions

## Introduction

Nervous system regulates homeostasis and adaptation to environmental changes of a whole organism, thus deregulation of nervous processes accelerates aging (Alcedo et al., 2013a,b). The aging process in different models is associated with progressive degeneration of the nervous system (Lee et al., 2000) and progression of age-related neurodegenerative diseases such as Alzheimer's disease, Parkinson's disease, Huntington's disease, and amyotrophic lateral sclerosis (Boerrigter et al., 1992; Coppede and Migliore, 2010). The neurodegeneration also characterizes the progeroid syndromes, including Hutchinson-Gilford syndrome and Werner's syndrome (Coppede and Migliore, 2010).

*Drosophila melanogaster* is a good model organism to study age-related neurodegenerative changes (Lu and Vogel, 2009). Enrichment in mutants with neurodegeneration among flies with shortened lifespan has been reported (Buchanan and Benzer, 1993; Kretzschmar et al., 1997). The brain from old flies demonstrates the ultrastructural neurodegenerative changes such as reduction in the number of synapses, defects in mitochondria, and increase in neuronal apoptosis (Haddadi et al., 2014). However, anti-aging interventions may postpone the neurodegeneration (Bgatova et al., 2015).

Here we consider molecular genetic changes in the *Drosophila* aging brain and the bases for applying the brain as a target for anti-aging intervention.

## Aging of the nervous system

The study of age-related gene transcriptional levels changes in *Drosophila* showed that in different organs (including the brain) there are two critical time points—30 and 60 day of age (Zhan et al., 2007). Comparing those points with *Drosophila* mortality curve it could be mentioned that the 30 day time point can be potentially attributed to the age when almost “linear” part of survival curve is followed by the “exponential” part, reflecting more rapid decrease the amount of live flies. These data are in good agreement with the shape of Gompertz curve, which describes the probability of age-related mortality in *Drosophila*. Gompertz curve has two parameters: R describes background mortality and α—exponential growth of mortality. At the initial 30 day of age Gompertz curve is close to the linear dependence with the R slope, at later 60 day of age the curve decrease exponentially. Our study of normal expression of *D-GADD45* gene during aging showed that *D-GADD45* brain expression is vanishing at critical point of 30 day old (Bgatova et al., 2015).

What are the genes that change the expression level during brain aging? As it is shown in (Girardot et al., 2006) the main effect is down regulation of genes involved in synaptic transmission at different levels divided into three subgroups. The first one includes genes that play a role in neurotransmitter metabolism such as the choline acetyltransferase (*Cha*) and the dopamine N acetyltransferase (*Dat*) genes. In the second subgroup many genes involved in various steps of neurotransmitter secretion: priming for synaptic vesicle fusion (γ-SNAP, unc13, comatose and tomosyn), fusion with presynaptic membrane (*Csp*, *Syx1A* and *rab3-GAP*) and reformation of vesicles through endocytosis (*liquid facets*, *AP-50* and *AP-2σ*). The third subgroup includes several neurotransmitter receptor ion channels. Among these channels, two nicotinic acetylcholine receptors (*nAcRβ 96A* and *nAcRα18C*) and three ionotropic glutamate receptors (*Nmdar1*, *GluCla*, and *CG11155*) mediate excitatory synaptic transmission. Moreover, three inhibitory GABAergic channels (*Lcch3*, *GABA-B-R2*, and *Rdl*) are also down regulated in aging *Drosophila* brain. Up regulated genes in aging *Drosophila* brain mostly present signatures similar to those observed in whole flies: genes associated with immune response and amino acid metabolism are over-represented. Based upon those whole genomic data it is possible to develop a set of Gal4 reporters that would permit to determine “biological” brain age markers for a given individual and to understand are there a “schedule” of aging at the gene level or is partially “stochastic” process.

## Nervous system as a target for anti-aging interventions

Genetic manipulations with a single gene expression that extend life span are important tools for discovering mechanisms underlying aging. Mutations in the *Indy* (I'mNotDeadYet) gene dramatically extend the lifespan of the fruit fly, *Drosophila melanogaster* (Rogina et al., 2000). In the past we had identified an allele *Indy-P115*, which shows the same life span extension as the first allele (Bulgakova et al., 2002, 2004). Since we had the *P(lArB)* insertion, we studied the pattern of expression of the gene in the larval tissue. It occurs that the larval brain has clear pattern of expression and we put forward a hypothesis that *Drosophila* brain can be the main target of aging.

Studies on other models confirm our assumption. For example, mutations in *daf-2* disrupting an insulin-like signaling pathway dramatically extend the adult *C. elegans* life span (Guarente and Kenyon, 2000). The study of cell-specificity of *daf-2* action reveals that the neurons are responsible for the effect (Wolkow et al., 2000). The *lit* mutant mouse strain, which has a mutation disrupting the hypothalamic GH releasing hormone (GHRH), lives longer. Homozygous *lit/lit* mice live up to 25% longer than wild-type mice (Flurkey et al., 2001).

The creation of Gene-Switch Gal4 drivers (Osterwalder et al., 2001) now permits to identify the genes, whose ectopic activation/suppression can prolong *Drosophila* life span when overexpressed in adults. In particular *Elav-GS* driver directs conditional RU486 expression in the nervous system. With this approach it was shown, that overexpression of *Cbs*, *Eip71CD*, *G6PD*, *GCLc*, *hep*, *Jafrac1*, *p53*, *Sir2* and the silencing of *CG9172*, *CG18809*, *l(3)neo18*, *Naam* in the adult brain leads to increased life span (Table 1). It is also necessary to mention that similar data was published for *D-Gadd45* (Plyusnina et al., 2011). Those data gave the heavy background to consider adult brain as the target of aging. However, the range of the genes tested with the approach is very small, so we like to analyze how large the range of such genes can be. All the genes mentioned above showed not only the life-span extension induced by *Elav-GS* driver, but similar extensions were observed also with one of *Act-GS-Gal4* or *Tub-GS-Gal4* drivers, showing ubiquitous over-expression also results in the life extension. So in Table 1 we made an attempt to correlate the list of the genes already studied by Gene-Switch approach with the level of their expression in development and tissues (modENCODE Tissue Expression Data). It can be seen that 30 genes studied within the *da-Gal4*, *tub-GS-Gal4*, *Act-GS-Gal4*, *hs-Gal4 UAS-geneX* system are heterogeneous group including high and low expression genes. Among those only *AGBE*, *CalpA*, *Men*, *wdb* demonstrate evident preponderance of head expression level. It is very probable that those genes, preferentially expressed in the head, also affects adult life-span by targeting the brain.

**Table 1:** **Tissue expression data of longevity genes in normal conditions**.

**GeneX**	**Expression pattern according to modENCODE Tissue Expression Data**	**References**
***da-Gal4, tub-Gal4, Act-Gal4, hs-Gal4 > UAS-GeneX***		
*Atg8a*	Very high expression almost in all organs including nervous system	Simonsen et al., 2008
*AGBE*	Moderately high, high in the head. Expression is lower in the other organs	Paik et al., 2012
*CalpA*	Moderate expression in the head. Expression is lower in the other organs	Paik et al., 2012
*CG8155*	Low expression in the head. Expression in other organs also low	Paik et al., 2012
*CG10383*	Low expression in the head. Expression in other organs also low	Paik et al., 2012
*CG10916*	Low expression in the head. Expression in other organs also low	Paik et al., 2012
*CG30427*	Low expression in the head. Expression in other organs also low	Paik et al., 2012
*CG42663*	Low expression in the head. Expression in other organs also low	Paik et al., 2012
*dFh*	Very low and low expression	Runko et al., 2008
*Dlc90F*	Moderately high, moderate expression in the head. Expression in other organs also high	Paik et al., 2012
*dPrx5*	Very high in testis, high and moderately high in other organs except salivary gland and fat body	Radyuk et al., 2009
*dTsc1*	High and moderately high in imaginal discs, ovary, and testis, moderate in almost all other organs	Gao et al., 2002
*GCLm*	High and very high expression in many organs, moderate expression in nervous system of larvae and pupae	Orr et al., 2005
*Hsp22*	Low expression in the head. Expression in other organs stronger	Kim et al., 2010
*Hsp26*	Low expression in the head. Expression in other organs stronger	Wang et al., 2004
*Hsp27*	Low expression in the head. Expression in other organs stronger	Wang et al., 2004
*ImpL2*	Moderately high, moderate expression in the head. Expression in other organs also high.	Paik et al., 2012
*Men*	High expression in the head. High expression in some other organs	Paik et al., 2012
*Nlaz*	High expression in the head. High expression in some other organs	Hull-Thompson et al., 2009
*Pcmt*	High expression in imaginal discs and testis, moderate expression in other organs including nervous system	Chavous et al., 2001
*PGRP-LF*	Weak expression everywhere	Paik et al., 2012
*Prx5*	High and moderately high in the head. High in other organs	Radyuk et al., 2009
*S6k*	Moderate, moderately high in the head and other organs	Kapahi et al., 2004
*SIFaR*	Low expression everywhere except pupae nervous system	Paik et al., 2012
*Sin3A*	Moderate in the head and some other organs	Paik et al., 2012
*sm*	Moderately high, moderate in the head. Expression higher in some organs	Paik et al., 2012
*Sod2*	High and very high in the head and other organs	Curtis et al., 2007
*Tor*	Low expression everywhere	Kapahi et al., 2004
*Trx-2*	Moderately high, moderate in the head and other organs	Seong et al., 2001
*Tsc1*	Moderate in the head and other organs	Kapahi et al., 2004
*wdb*	Moderately high, moderate in the head and some other organs	Funakoshi et al., 2011
***da-Gal4, tub-Gal4 > RNAi-GeneX***		
*CG17856*	Very low everywhere	Copeland et al., 2009
*ms(3)72Dt*	Very low everywhere	Copeland et al., 2009
***Elav-Gal4 > UAS-GeneX***		
*Cbs*	Low everywhere	Kabil et al., 2011
*Eip71CD*	High, moderately high in the head and some other organs	Chung et al., 2010
*G6PD*	Moderate expression in testis and head of 20 day male, moderate and low expression in other organs. Expression in nervous system of larvae and pupae—very low	Legan et al., 2008
*GCLc*	High expression in digestive system and salivary glands	Orr et al., 2005
*Hep*	Moderate in the head and other organs	Biteau et al., 2010
*Jafrac1*	Very high, high, and moderately high almost everywhere, exept salivary gland fat body	Lee et al., 2009
*p53*	Very low everywhere	Shen et al., 2009
*Sir2*	Moderate in the head and other organs	Whitaker et al., 2013
***Elav-Gal4 > RNAi-GeneX***		
*CG9172*	High, moderately high in the head and some other organs	Copeland et al., 2009
*CG18809*	Moderately high, moderate in the head. Expression lower in other organs	Copeland et al., 2009
*l(3)neo18*	Very high in the head and some other organs	Copeland et al., 2009
*Naam*	Very low in the head and other organs	Balan et al., 2008

It was discovered cases when ubiquitous drivers: *da-GS-Gal4* and *tub-GS-Gal4* can extend life-span when inducing *RNAi-geneX* constructs (Table 1). Among those only *CG17856*, *ms(3)72Dt* have very low level of expression in the head.

Recent investigations shown, that the nervous system may be a target for ant-aging pharmacological interventions also. For example, serotonin antagonists (272N18, mianserin, mirtazapine, methiothepin and cyproheptadine), some of which are used clinically, extend the lifespan of adult *C. elegans* by 20–33% (Petrascheck et al., 2007). Screening of a library of compounds with known mammalian pharmacology revealed 60 compounds that increase longevity in *C. elegans* (Ye et al., 2014). The 33 compounds increased resistance to oxidative stress, and enhanced resistance to oxidative stress was associated primarily with compounds that target receptors for biogenic amines, such as dopamine or serotonin (Ye et al., 2014).

## Conclusion

Now the thesis “*Drosophila* nervous system as a target of aging and anti-aging interventions” has been proved for some cases. On the one side of the nervous system is one of the targets of aging process and the state of nervous system may be regarded as a marker of aging. In this context, intervention aimed to combat the aging should lead to postponement of neurodegeneration. On the other hand, many pharmacological and genetic aging-suppressive interventions act through the nervous system. Therefore, it can be considered as one of the targets of anti-aging therapy. However, conditional expression approach reveals also other essential targets. We think that now days, when a large list of longevity genes already become known, it needs to put some efforts for complete longevity targets determination for every case. For example, current studies of the *Indy* mutations extending life-span are concentrated on the gene function in the gut (Rogina et al., 2014). However, for the most of the longevity genes the target organs are poorly studied. We suggest that the brain is one of the main aging targets.

### Conflict of interest statement

The authors declare that the research was conducted in the absence of any commercial or financial relationships that could be construed as a potential conflict of interest.
